# Loss of the *Drosophilam*-AAA mitochondrial protease paraplegin results in mitochondrial dysfunction, shortened lifespan, and neuronal and muscular degeneration

**DOI:** 10.1038/s41419-018-0365-8

**Published:** 2018-02-21

**Authors:** Gautam Pareek, Ruth E. Thomas, Leo J. Pallanck

**Affiliations:** Department of Genome Sciences, University of Washington, 3720 15th Avenue, NE, Seattle, WA 98195 USA

## Abstract

The progressive accumulation of dysfunctional mitochondria is implicated in aging and in common diseases of the elderly. To oppose this occurrence, organisms employ a variety of strategies, including the selective degradation of oxidatively damaged and misfolded mitochondrial proteins. Genetic studies in yeast indicate that the ATPase Associated with diverse cellular Activities (AAA^+^) family of mitochondrial proteases account for a substantial fraction of this protein degradation, but their metazoan counterparts have been little studied, despite the fact that mutations in the genes encoding these proteases cause a variety of human diseases. To begin to explore the biological roles of the metazoan mitochondrial AAA^+^ protease family, we have created a CRISPR/Cas9 allele of the *Drosophila* homolog of *SPG7*, which encodes an inner membrane-localized AAA^+^ protease known as paraplegin. *Drosophila SPG7* mutants exhibited shortened lifespan, progressive locomotor defects, sensitivity to chemical and environmental stress, and muscular and neuronal degeneration. Ultrastructural examination of photoreceptor neurons indicated that the neurodegenerative phenotype of *SPG7* mutants initiates at the synaptic terminal. A variety of mitochondrial defects accompanied the degenerative phenotypes of *SPG7* mutants, including altered axonal transport of mitochondria, accumulation of electron-dense material in the matrix of flight muscle mitochondria, reduced activities of respiratory chain complexes I and II, and severely swollen and dysmorphic mitochondria in the synaptic terminals of photoreceptors. *Drosophila SPG7* mutants recapitulate key features of human diseases caused by mutations in *SPG7*, and thus provide a foundation for the identification of *Drosophila* paraplegin substrates and strategies that could be used to ameliorate the symptoms of these diseases.

## Introduction

Mitochondria have essential cellular roles in ATP synthesis, calcium homeostasis, and metabolism, but these activities come at a cost. In particular, the production of ATP through oxidative phosphorylation leads to the generation of reactive oxygen species (ROS) that can damage mitochondrial proteins, lipids, and DNA^1,2^. Moreover, the respiratory chain complexes responsible for the production of ATP require the coordinated expression of mitochondrial and nuclear encoded subunits, and an imbalance in the stoichiometry of these subunits can result in protein misfolding and aggregation. The progressive accumulation of oxidatively damaged and misfolded mitochondrial proteins is strongly implicated in aging and common diseases of the elderly, including neurodegenerative diseases and cancer^1–3^_._ To oppose the accumulation of dysfunctional mitochondria, metazoans employ a variety of quality control strategies, including the selective degradation of dysfunctional mitochondria or their damaged components^4,5^. Although the recent identification of the mitophagy-promoting factors PTEN-induced putative kinase 1 (PINK1) and Parkin has led to rapid advancement in our understanding of mitochondrial degradation, comparatively less is known of the mechanisms by which damaged mitochondrial components are detected and degraded^6–8^.

Previous work suggests that the AAA^+^ family of mitochondrial proteases have a major role in mitochondrial quality control by degrading oxidatively damaged and misfolded proteins^9,10^. There are four major mitochondrial AAA^+^ proteases in metazoans including the inner membrane-localized proteases *i*-AAA and *m*-AAA, and the matrix-localized proteases LON and Clp^9^. The catalytic domains of the *m*-AAA and *i*-AAA proteases face the matrix and intermembrane space, respectively^10^. All four of these proteases form multimeric protein complexes that use energy derived from ATP hydrolysis to unfold and transport substrates to an internal proteolytic domain for degradation. Lon and *i*-AAA assemble as homo-oligomeric complexes, whereas Clp is composed of proteolytic (ClpP) and ATPase (ClpX) subunits. The *m*-AAA protease comes in two forms: hetero-oligomeric complexes of the paraplegin protein and the ATPase family gene 3-like 2 (AFG3L2) protein, and homo-oligomeric complexes of AFG3L2^9,10^. The importance of these proteases is illustrated by the fact that mutations in the genes encoding them cause a variety of human diseases, including hereditary spastic paraplegia (HSP), spinocerebellar ataxia type 28, and perrault syndrome^9–13^. However, the substrates of these proteases, and the mechanisms by which mutations in the genes encoding them cause disease, are largely unknown.

To begin to explore the biological roles of the AAA^+^ mitochondrial protease family, we have created a CRISPR/Cas9 deletion allele of one of the *Drosophila m*-AAA family members, *SPG7*, which encodes a homolog of paraplegin. We found that *SPG7* mutants display shortened lifespan, locomotor impairment, sensitivity to stressors, and degeneration of the indirect flight muscle (IFM) and the nervous system. These phenotypes were accompanied by mitochondrial trafficking defects in the nervous system, an accumulation of swollen mitochondria containing electron-dense aggregates, and significantly reduced activity of respiratory chain complexes I and II. Transmission electron microscopic analysis of photoreceptor neurons revealed severe architectural alterations restricted to the synaptic terminals, thus recapitulating the synaptopathy and axonopathy associated with HSP^10^_._ Our work provides a foundation to apply the powerful genetic tools of *Drosophila* to study the mechanisms underlying the neurological diseases associated with *SPG7* mutations in humans^10,14–16^.

## Results

### Identification of a *Drosophila* paraplegin homolog

**Fig. 1:**
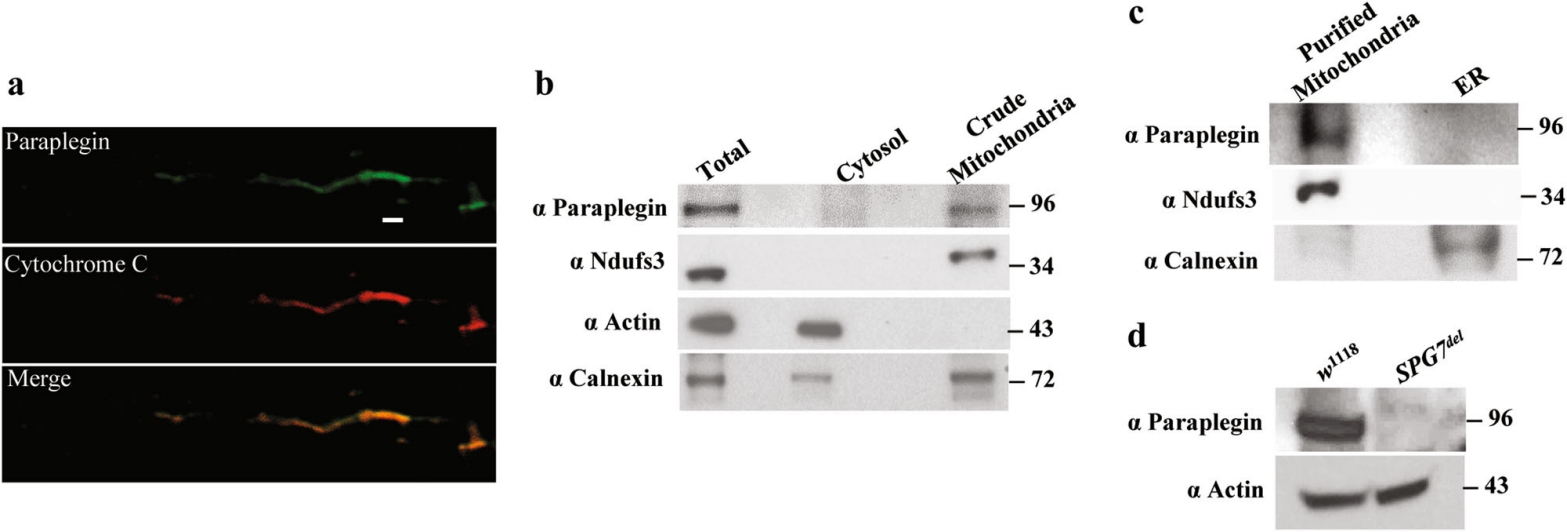
*Drosophila* paraplegin localizes to mitochondria. **a** Confocal images of indirect flight muscles using anti-paraplegin (upper panel) and anti-cytochrome *c* (middle panel) antisera. The merged image (lower panel) indicates that paraplegin and cytochrome *c* colocalize. Scale bar is 1 μm. **b** Mitochondrial and cytosolic fractions were isolated by differential centrifugation from adult flies and immunoblotted using antisera against paraplegin, Ndufs3 (mitochondria), Actin (cytosol), and Calnexin (endoplasmic reticulum) showing that paraplegin localizes to the mitochondrial fraction. **c** Purified mitochondrial and endoplasmic reticulum (ER) fractions produced from sucrose gradient sedimentation of a crude mitochondrial fraction were subjected to western blotting using antisera to paraplegin, Ndufs3 (mitochondria) and Calnexin (endoplasmic reticulum). Results indicate that paraplegin localizes to mitochondria, not ER. **d** Western blot analysis of *w*^1118^ and *SPG7*^*del*^ mutants using an anti-paraplegin antiserum and an antiserum to actin as a loading control. Note that the band corresponding in size to the mature paraplegin protein is absent in *SPG7*^*del*^ mutants

To identify a *Drosophila* paraplegin homolog, we used the human paraplegin protein sequence to conduct a Basic Local Alignment Search Tool (BLAST) search, and identified three *Drosophila* genes encoding proteins with 43–58% identity to human paraplegin. Two of these genes, *CG6512* and *CG3499*, encode the previously characterized mitochondrial AAA^+^ protease family members Afg3l2 and dyme1l, respectively^10,17^. The third gene, *CG2658*, encodes a previously uncharacterized homolog of human paraplegin with 58% identity and 75% similarity at the protein level (Supplementary Figure S1A). Data from the *Drosophila* modENCODE and FlyAtlas data repositories indicate that the *CG2658* gene is ubiquitously expressed in all tissues throughout development. The Pfam motif prediction algorithm predicts that the polypeptide encoded by *CG2658* contains an AAA domain (amino acid residues 377–513) and an M41 metallopeptidase domain (amino acid residues 575–785) acting as a proteolytic center (Supplemental Figure S1B). The MitoProt algorithm predicts that the N-terminal region of paraplegin contains a mitochondrial targeting sequence, and a proteomic proximity ligation study detected peptides corresponding to *CG2658* in the mitochondrial matrix^18^. We generated an antiserum to the *CG2658* gene product and used this antiserum to confirm the mitochondrial localization of this protein (Fig. 1a–c). Given these findings, and the fact that *CG2658* encodes the most closely related protein to paraplegin, we propose that this gene represents the *Drosophila* ortholog of *SPG7*.

### An *SPG7* null mutant exhibits shortened lifespan and behavioral abnormalities

To explore the biological role of *SPG7*, we used the CRISPR/CAS9 system to replace the entire *SPG7* coding sequence with the DsRed marker _(_Supplementary Figure S2)^19^. Flies with DsRed expression were subjected to whole-genome sequencing to verify the successful creation of an *SPG7* deletion (*SPG7*^*del*^), and males hemizygous for this deletion were subjected to western blot analysis to confirm that *SPG7*^*del*^ represents a null allele (Fig. 1d).These flies were then backcrossed six times to an isogenic *w*^1118^ stock to allow direct phenotypic comparison with a control stock.

**Fig. 2:**
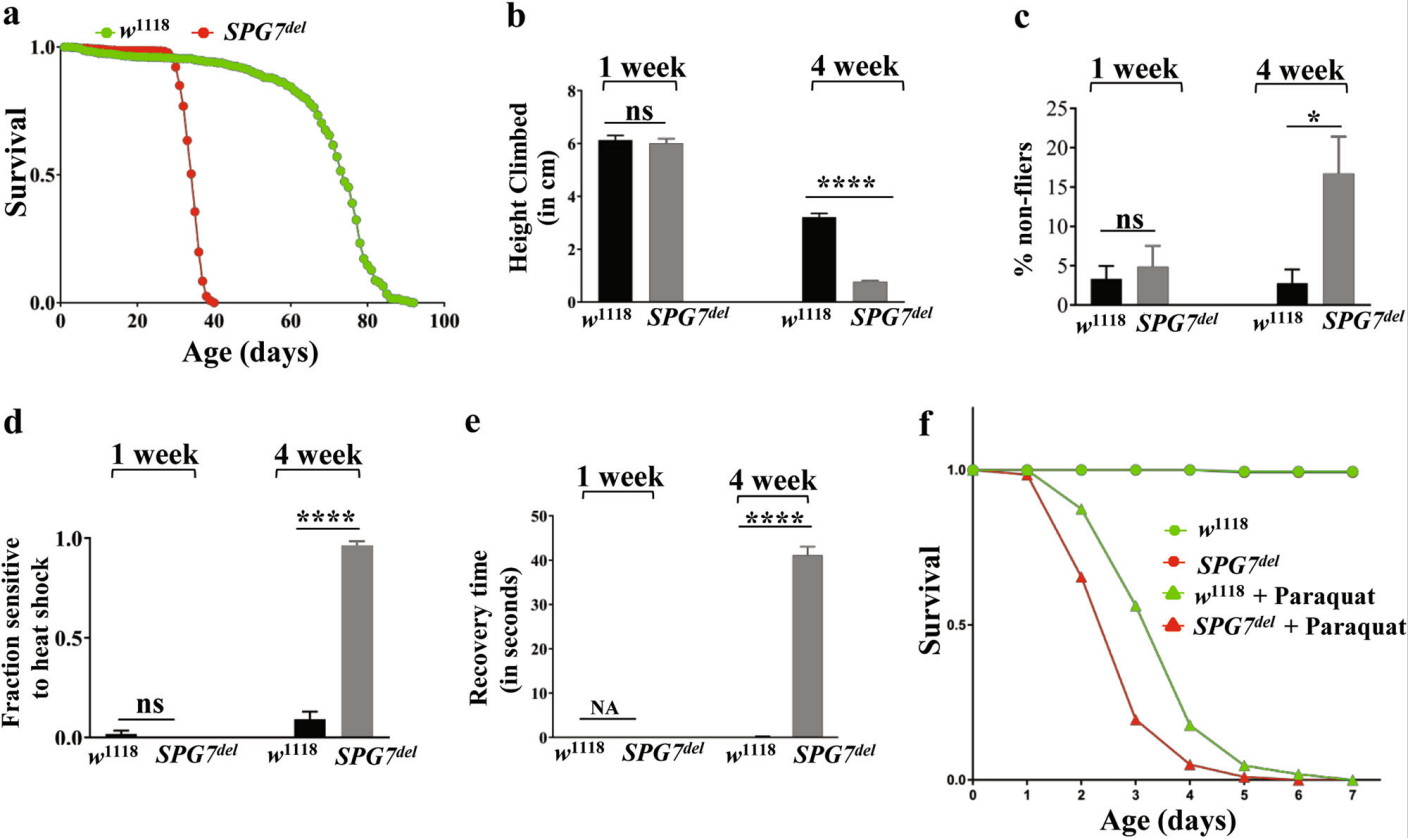
Shortened lifespan and behavioral abnormalities in *SPG7*^*del*^ mutants. **a** Kaplan–Meier survival curves of *SPG7*^*del*^ flies (*SPG7*^*del*^, *N* = 671, 50% survival 34 days) and controls (*w*^1118^, *N* = 752, 50% survival 74 days). Significance was determined using a log-rank test (*p* < 0.0005). **b** *SPG7*^*del*^ mutants exhibit an age-dependent climbing defect. Error bars represent s.e.m. (*N* = 200 for each genotype, ****p < 0.0005 by Student’s *t*-test). **c** *SPG7*^*del*^ mutants exhibit a significant increase in the fraction of flies that are incapable of flight. The histograms represent the fraction of non-fliers of the indicated genotypes, and error bars represent the s.e.m. (*n* = 6 independent groups of 10–15 animals, *p < 0.05 from Student’s *t*-test). **d** *SPG7*^*del*^ mutants are sensitive to heat-induced paralysis. The histograms depict the percentage of flies sensitive to heat shock. Error bars represent s.e.m. (*N* = 55 for *w*^1118^ and *N* = 83 for *SPG7*^*del*^ flies, ****p < 0.0005 by Mann–Whitney *U*-test). **e** *SPG7*^*del*^ mutants exhibit mechanical stress-induced paralysis. Recovery time from mechanical stress (bang sensitivity) is shown for flies of the indicated genotype and age. Error bars represent s.e.m. (*N* = 91 for each genotype, ****p < 0.0005 from Student’s *t*-test). **f** *SPG7*^*del*^ flies are more sensitive to exposure to the oxidative stress-inducing agent paraquat (*n* = 200, ****p < 0.0005 by log-rank test)

*SPG7*^*del*^ mutants were viable and fertile, with no detectable anatomical or behavioral phenotypes at a young age. However, they were significantly shorter-lived than wild-type flies, with a median survival of 34 days compared to 73 days for control flies (Fig. 2a). Because premature aging in *Drosophila* is frequently associated with defective locomotor performance, we tested whether the shortened lifespan of *SPG7*^*del*^ mutants was accompanied by climbing or flight defects. To test climbing ability, we tapped flies to the bottom of a vial and measured the distance climbed in a given interval of time. Flight ability was measured by releasing flies into the top of a graduated cylinder and noting where flies alight^20^. Strong fliers land near the top of the cylinder, whereas weaker fliers land lower in the cylinder. The climbing assay detected a severe climbing defect in *SPG7*^*del*^ mutants at 4 weeks of age (Fig. 2b). *SPG7*^*del*^ mutants also displayed a progressive biphasic flight defect: at 4 weeks of age, most flies landed near the top of the cylinder, but a small but significant fraction fell all the way to the bottom of the cylinder (Fig. 2c). The lifespan and locomotor phenotypes of *SPG7*^*del*^ mutants were rescued by autosomes containing short duplications spanning the *SPG7* gene (Supplemental Figure S3A), providing evidence that these phenotypes are a consequence of the *SPG7* deletion.

Many *Drosophila* mutants that bear defects in genes encoding mitochondrial proteins exhibit stress sensitivity^21^. Thus, we tested the sensitivity of *SPG7*^*del*^ mutants to three commonly used stressors: high temperature, mechanical stimulation (bang sensitivity), and exposure to an oxidative stress agent. The first two of these stressors can lead to paralysis in sensitive backgrounds, whereas the third can exacerbate the shortened lifespan of a mitochondrial mutant^17^. Exposing *SPG7*^*del*^ mutants to 39 °C for 6 min resulted in a progressive age-dependent heat intolerance compared to wild-type controls (Fig. 2d). The *SPG7*^*del*^ mutants also exhibited a progressive and severe bang-sensitive phenotype (Fig. 2e), and 4-week-old *SPG7*^*del*^ mutants displayed seizure-like behavior with regular rhythmic wing and leg jerking even in the absence of mechanical stress (data not shown). Finally, *SPG7*^*del*^ flies exhibited enhanced sensitivity to the oxidative stress-inducing agent paraquat (Fig. 2f). Together, these findings establish that *SPG7*^*del*^ mutants are sensitive to a wide range of environmental stresses.

### *SPG7*^*del*^ mutants exhibit muscular and neuronal degeneration

**Fig. 3:**
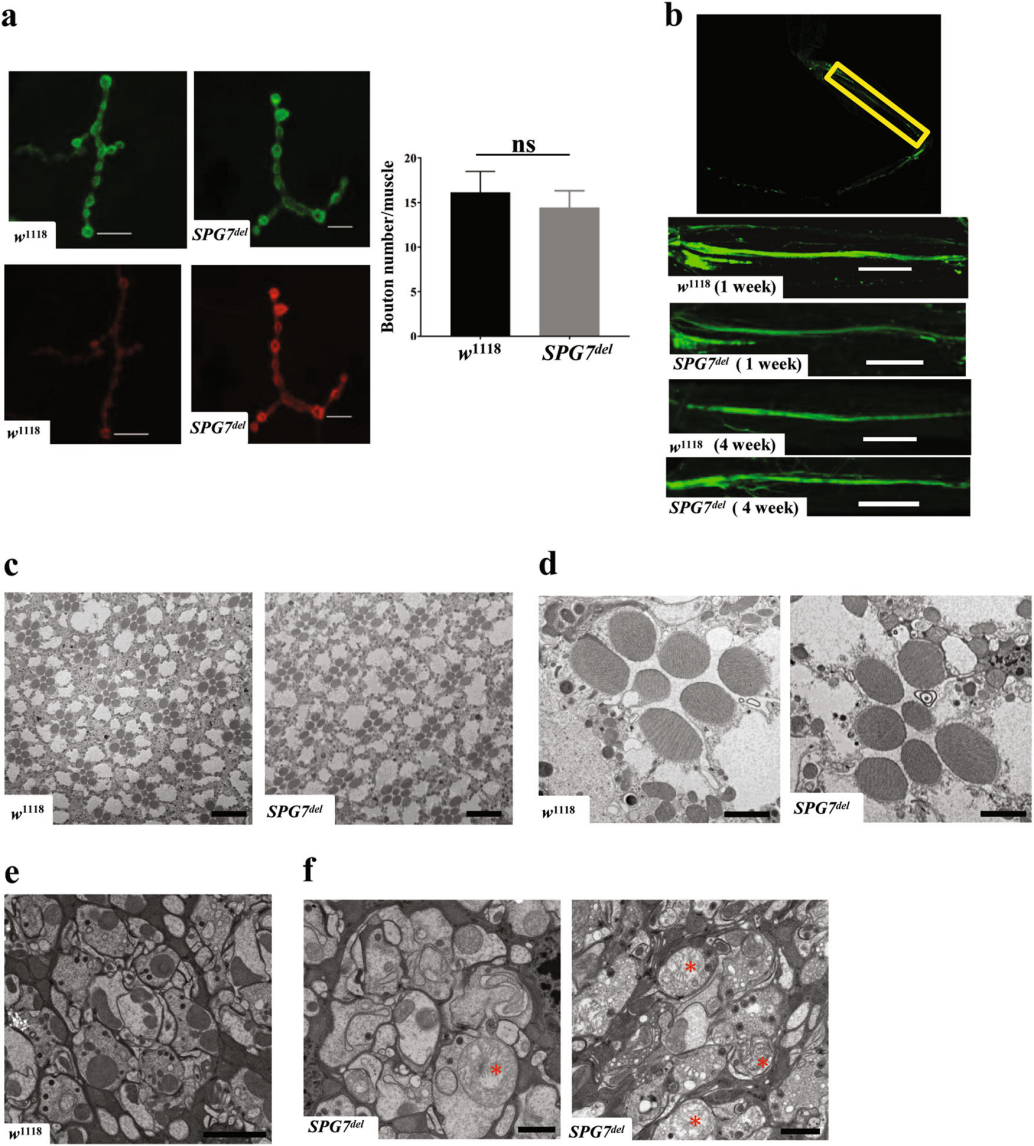
Axonal and synaptic defects in *SPG7*^*del*^ mutant flies. **a** Immunostaining of the neuromuscular junction from muscle 4 of third instar larvae of the indicated genotypes using anti-discs large 1 (upper panel) and anti-HRP (lower panel) antisera as postsynaptic and presynaptic markers, respectively. Right panel shows quantification of bouton numbers in the two genotypes (*n* = 10). Significance was determined using Student’s *t*-test. **b** Motor neurons were visualized in the femur region (highlighted in yellow box) from adult fly legs using the neuronal marker mCD8-GFP expressed using the *D42-GAL4* driver from 1-week-old and 4-week-old *w*^1118^ control and *SPG7*^*del*^ mutants (*n* = 20). Scale bar is 10 μm. **c** Representative transmission electron micrograph (TEM) images of photoreceptors from 4-week-old *w*^1118^ controls and *SPG7*^*del*^ mutants. A single ommatidium is shown in **d** from *w*^1118^ control and *SPG7*^*del*^ mutants. **e** TEM micrograph of lamina cartridge from 4-week-old *w*^1118^ control. **f** TEM micrographs of single lamina cartridges from 4-week-old *SPG7*^*del*^ mutant. Note the presence of swollen mitochondria with aberrant cristae structure (red asterisks), and the disorganized appearance of photoreceptor terminals in *SPG7*^*del*^ mutants, relative to controls. Scale bar for **c** 10 μm, for **d** 2 μm and for **e**, **f** 1 μm

Mutations in human *SPG7* cause the neurological disorder hereditary spastic paraplegia, a distal axonopathy characterized by the loss of corticospinal motor neurons, and deletion of the *SPG7* gene in mice also results in axonal and synaptic alterations^22,23^. Thus, we tested whether the behavioral phenotypes of *SPG7*^*del*^ mutants arise from similar neuronal defects. To evaluate whether *SPG7*^*del*^ influences synapse formation or maintenance, we quantified the number of type Ib synaptic boutons in larval body wall muscle 4 segments A2 and A3 using presynaptic (anti-HRP) and postsynaptic (anti-discs large 1) markers^24,25^. We also used the motor neuron-specific driver *D42-Gal4* to label motor neurons in adult fly legs with GFP, and analyzed axonal morphology in young (1 week) and old animals (4 week) using confocal microscopy^26^. Finally, we examined the integrity of photoreceptor neurons in 4-week-old animals using transmission electron microscopy (TEM)^27^. We did not detect alteration in the number of synaptic boutons in *SPG7*^*del*^ third instar larvae (Fig. 3a), axonal loss in motor neurons in the adult leg (Fig. 3b), or reduction in the number of photoreceptor neurons in the visual system (Fig. 3c, d). However, in contrast to the well-organized photoreceptor synaptic terminals in wild-type flies, the synaptic terminals of *SPG7*^*del*^ mutants were disorganized and showed accumulation of swollen and morphologically abnormal mitochondria (Fig. 3e, f)^27,28^. Together, these studies indicate that paraplegin influences synapse integrity in a cell type-dependent manner.

**Fig. 4:**
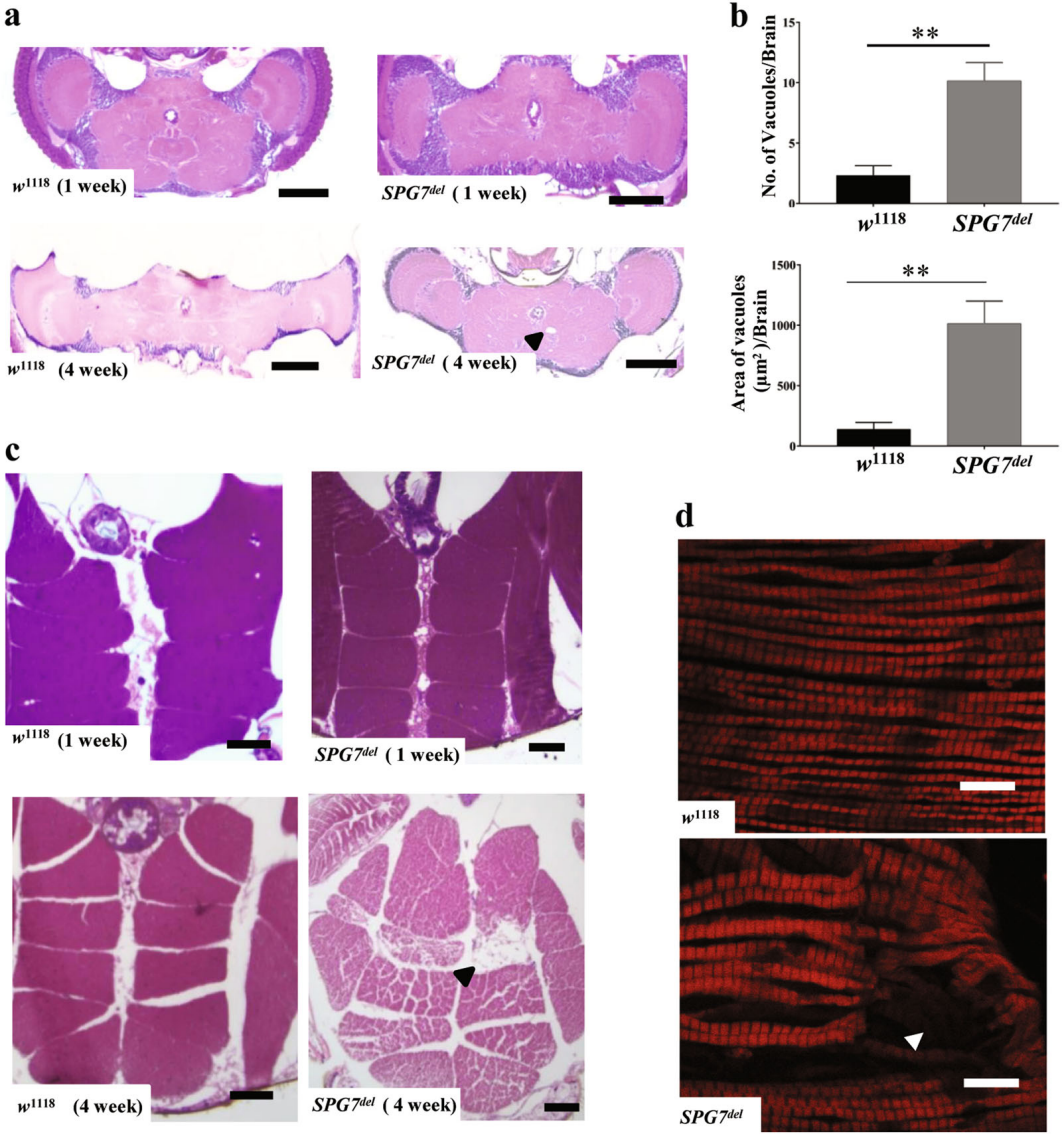
Loss of paraplegin affects tissue integrity. **a** Brain integrity of *w*^1118^ control and *SPG7*^*del*^ mutants was analyzed by hematoxylin- and eosin-stained, paraffin-embedded brain sections at 1 week and 4 weeks of age. The black arrowhead indicates a vacuole in *SPG7*^*del*^ mutants. Scale bar is 100 μm. **b** Quantification of brain vacuole number (upper panel) and area (bottom panel) in 4-week-old flies of the indicated genotype (*n* = 6, **p < 0.005 from Student’s *t*-test). **c** Representative images of thoracic cross sections from *w*^1118^ control and *SPG7*^*del*^ mutants at 1 week and 4 weeks age. Degenerating muscle tissues are indicated by the black arrowhead. Scale bar is 50 μm. **d** Confocal imaging of indirect flight muscles from 4-week-old *w*^1118^ control and *SPG7*^*del*^ mutants stained using the actin-binding compound phalloidin. Degenerating muscle fibers are highlighted by white arrowhead. Scale bar is 10 μm

Although the alterations in photoreceptor terminals demonstrate that paraplegin is critical for synaptic integrity, a synaptic defect confined to the visual system cannot explain the behavioral phenotypes of *SPG7*^*del*^ mutants. To further explore the origin of these deficits, we performed histological analysis of paraffin-embedded brain and thorax sections. Brain sections from old *SPG7*^*del*^ mutants revealed a significantly increased number of vacuoles relative to age-matched controls (Fig. 4a, b). The vacuoles were present throughout the brain, more frequently in the central neuropil region than in the optic lobes, indicating that loss of paraplegin causes progressive deterioration of brain tissue in *Drosophila* (Fig. 4a). Transverse thoracic sections also revealed a progressive loss of the integrity of IFMs in *SPG7*^*del*^ mutants, consistent with the essential metabolic role of mitochondria in flight muscle maintenance (Fig. 4c). However, this phenotype was only partially penetrant (data not shown), and we therefore used a second assay to verify the findings. Phalloidin staining of indirect flight muscles confirmed the muscle degeneration phenotype of old *SPG7*^*del*^ mutants (Fig. 4d). Together, our findings demonstrate an essential role for *SPG7*^*del*^ in neuronal and IFM integrity.

### *SPG7*^*del*^ mutants accumulate morphologically and functionally abnormal mitochondria

**Fig. 5:**
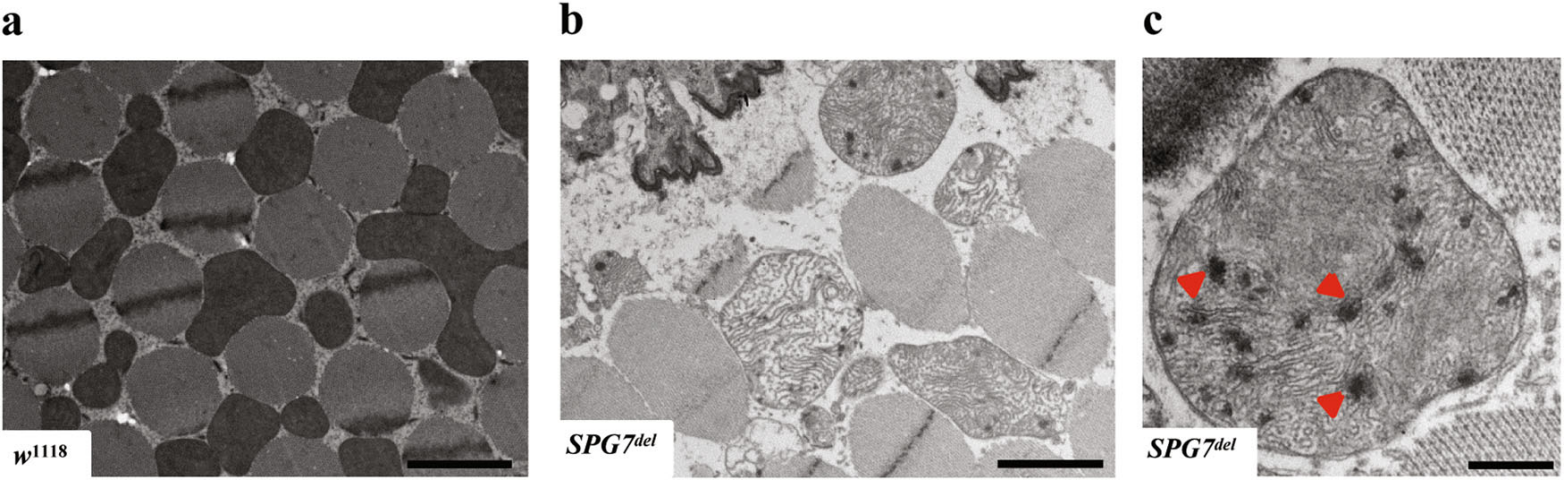
Mitochondrial morphological abnormalities in indirect flight muscles of *SPG7*^*del*^ flies. **a**–**c** Representative TEM images from indirect flight muscles of 4-week-old *w*^1118^ control (**a**) and *SPG7*^*del*^ (**b**, **c**) mutants. Note the presence of aberrant cristae and dense inclusions in mitochondria from *SPG7*^*del*^ mutants relative to controls (arrowheads in **c**). Scale bar is 2 μm in **a** and **b**, and 0.5 μm in **c**

The mitochondrial morphological defects in the photoreceptor terminals of *SPG7*^*del*^ mutants, and the known role of paraplegin in mitochondrial protein degradation, suggest that the neuronal and muscular degeneration phenotypes of *SPG7*^*del*^ mutants could derive from mitochondrial dysfunction. To explore this matter more fully, we performed several additional experiments. First, we investigated the ultrastructure of mitochondria in the IFMs using TEM in cross sections of thoraces^6^. Strikingly, IFMs from old *SPG7*^*del*^ mutants (4 week) showed large, swollen, and loosely packed mitochondria with disorganized cristae (Fig. 5). These abnormal mitochondria frequently contained electron-dense material that appeared to reside in the matrix (Fig. 5b, c). This finding is consistent with previously published reports on other AAA^+^ protease mutants^17,29,30^.

**Fig. 6:**
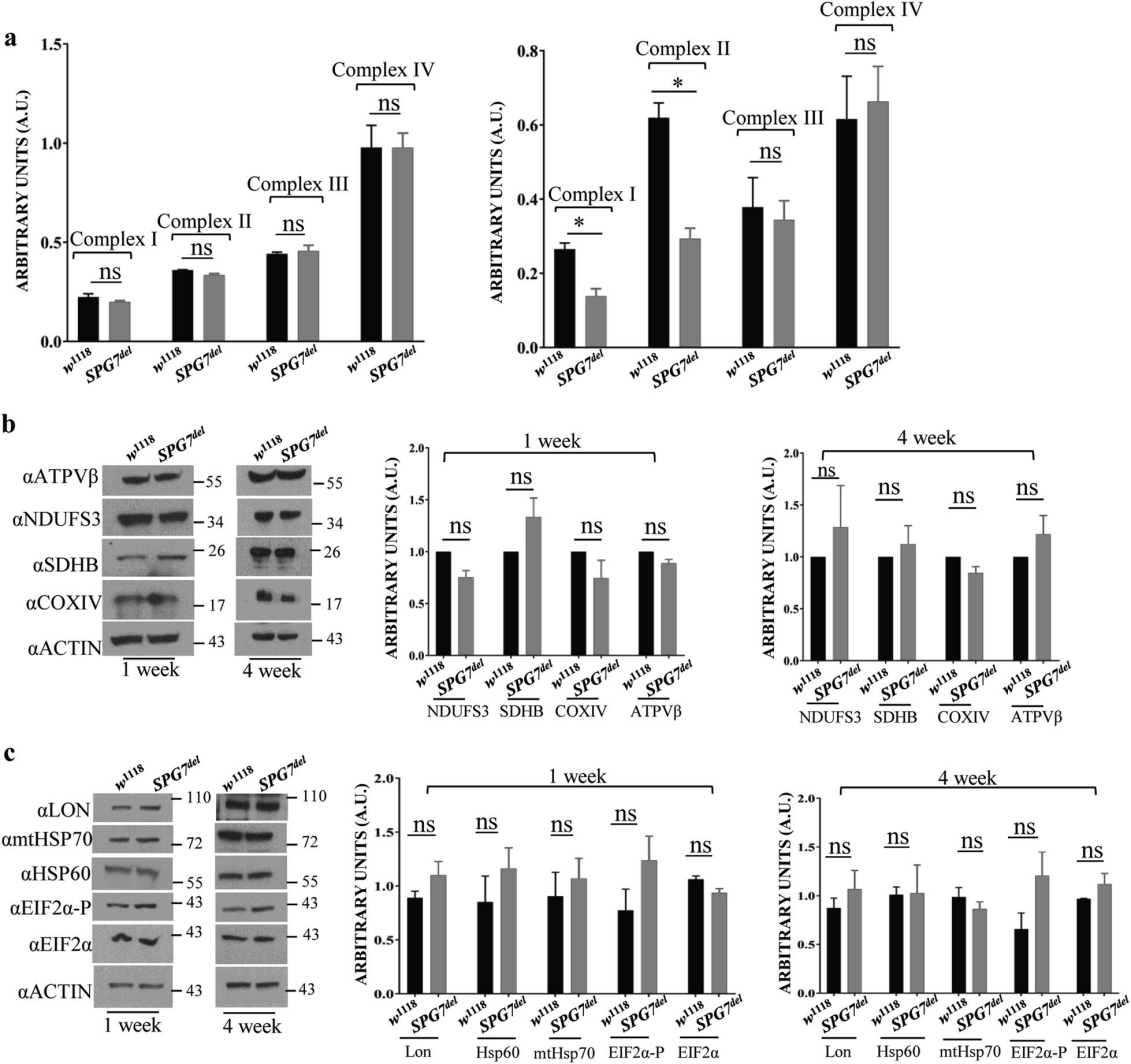
Mitochondrial respiratory chain dysfunction in *SPG7*^*del*^ flies. **a** The activity of respiratory chain complexes in *w*^1118^ control and *SPG7*^*del*^ mutants at 1 week (left panel) and at 4 weeks (right panel) of age. Aged *SPG7*^*del*^ mutants exhibit reduced complex I and II activity (*n* = 3, *p < 0.05 from Student’s *t*-test). **b** Results of western blot analysis to quantify the abundance of the indicated respiratory chain complex subunits in young (1 week; left panel) and old (4 week; right panel) *w*^1118^ control and *SPG7*^*del*^ mutants are shown. **c** Western blot analysis of UPR^mt^ markers in young (1 week) and old (4 week) *w*^1118^ control and *SPG7*^*del*^ mutants are shown

To test whether the mitochondrial morphological alterations detected in our TEM studies were also associated with compromised respiratory chain (RC) function, we used established assays to quantify RC activity^31^. RC complex activity was normal in young (1 week) *SPG7*^*del*^ mutants (Fig. 6a, left panel), but we observed significant decreases in the activities of complexes I and II in 4-week-old *SPG7*^*del*^ mutants relative to controls. However, the activities of complexes III and IV remained unaffected even in old *SPG7*^*del*^ mutants (Fig. 6a, right panel). To explore the mechanisms underlying the reduced complex I and II activity in old *SPG7*^*del*^ mutants, we analyzed the abundance of these complexes, as well as selected subunits of these complexes by immunoblotting. Blue native gels revealed an age-dependent decline in the abundance of assembled complex I in *SPG7*^*del*^ mutants (Supplemental Figure S4), but the abundance of all tested subunits of complex I was normal in both young and old *SPG7*^*del*^ mutants (Fig. 6b; Supplemental Figure S5). We also failed to detect a difference in the abundance of complex II subunits between *SPG7*^*del*^ mutants and controls (Fig. 6b; Supplemental Figure S5), but we were unable to detect fully assembled complex II upon blue native gel analysis, possibly because the epitope detected by the antiserum we used is inaccessible in the fully assembled complex. Our findings suggest that the decrease in the activity of complex I reflects an age-dependent defect in the assembly of complex I. The molecular basis of reduced complex II activity will require further analysis.

A failure to assemble or degrade misfolded mitochondrial proteins has been shown to activate the mitochondrial unfolded protein response (UPR^mt^) pathway, a nuclear response to mitochondrial stress that involves, among other things, induction of mitochondrial chaperones^32^. To test whether the electron-dense accumulations detected in IFM mitochondria activate the UPR^mt^, we examined the abundance of several different markers of this stress pathway, including Hsp60, mtHsp70, and Lon protease. We also examined the abundance of phosphorylated eIF2α, which serves to attenuate cytosolic translation in response to UPR^mt^^33^. Although we detected a trend towards increased abundance of phosphorylated eIF2α in both young and old *SPG7*^*del*^ flies (Fig. 6c), the differences were not statistically significant. In addition, none of the tested markers of UPR^mt^ showed significant induction (Fig. 6c). These findings indicate that, despite the electron-dense deposits in the matrix of muscle mitochondria of *SPG7*^*del*^ mutants, any accumulation of misfolded proteins is insufficient to trigger induction of the UPR^mt^. Further work will be required to explain the origin of the electron-dense matrix accumulations in *SPG7*^*del*^ mutants.

### Loss of *SPG7* causes mitochondrial trafficking defects in larval segmental nerves

**Fig. 7:**
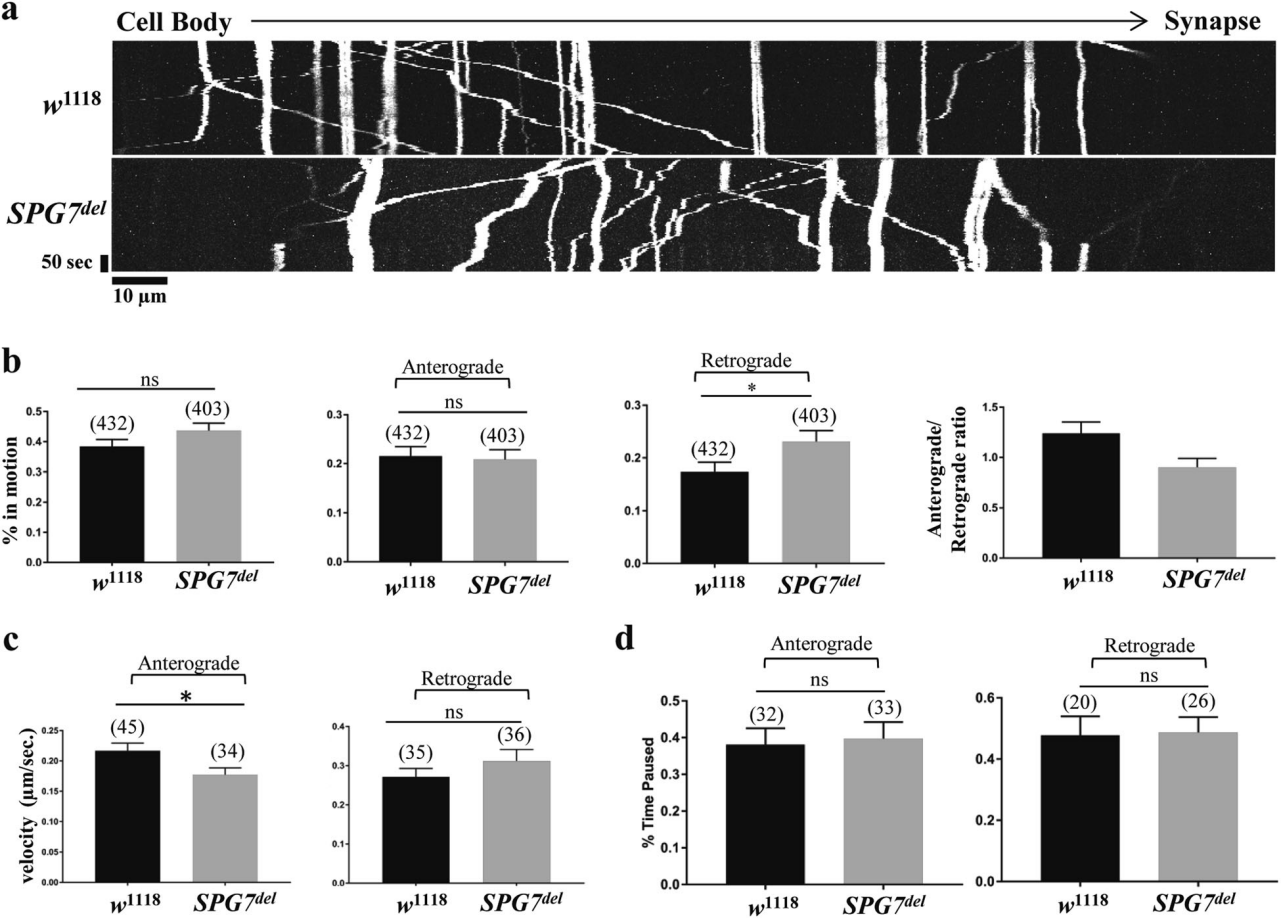
*SPG7*^*del*^ mutants exhibit increased retrograde transport of mitochondria. **a** Representative kymographs of axonal mitochondrial transport in larval segmental nerves of wild-type and *SPG7*^*del*^ larvae. Anterograde movement is left to right and retrograde movement is right to left in the images. Vertical lines in the kymographs indicate stationary mitochondria. **b** Loss of *SPG7* enhances net retrograde transport of mitochondria and decreases the anterograde/retrograde ratio (*p < 0.05 from Mann–Whitney *U*-test). **c** Loss of *SPG7* results in reduced anterograde mitochondrial velocity (*p < 0.05 from Student’s *t*-test). **d** *SPG7* does not influence the fraction of time mitochondria remain paused. Numbers in parenthesis in **b** and **c** indicate total number of mitochondria used for quantification. Significance was determined using Student’s *t*-test

Many loci associated with HSP encode factors that potentially influence mitochondrial trafficking. These findings, coupled with the mitochondrial phenotypes of *SPG7*^*del*^ mutants, led us to explore the influence of *SPG7* on axonal mitochondrial transport. We used the *Drosophila* larval segmental nerve to compare a variety of mitochondrial trafficking parameters in *SPG7*^*del*^ mutants (Supplementary Movie 2) and *w*^1118^ controls (Supplementary Movie 1). Loss of *SPG7* resulted in a significant increase in the fraction of mitochondria moving in the retrograde direction and an anterograde to retrograde transport ratio below 1 (Fig. 7a, b). We also found that the velocity of mitochondria moving in the anterograde direction was significantly decreased and there was a trend towards increased velocity of mitochondria moving in the retrograde direction in *SPG7*^*del*^ mutants (Fig. 7c). However, the overall levels of activity of mitochondrial motors were unchanged as determined by the fraction of time a moving mitochondria remain stationary (Fig. 7d). Together our findings indicate that loss of *SPG7* results in a net increase in the movement of mitochondria in the retrograde direction.

## Discussion

Mitochondrial dysfunction is a central hallmark of aging and a frequent occurrence in neurodegenerative disorders including Alzheimer’s disease, Parkinson’s disease, and Huntington’s disease^2–4^. Mitochondrial proteases represent one of the first lines of defense against mitochondrial dysfunction by promoting the degradation of damaged and misfolded mitochondrial proteins. The importance of mitochondrial proteases is illustrated by the fact that mutations in the genes encoding these proteases are the cause of multiple human diseases. In particular, mutations in *SPG7* have been implicated in cerebellar ataxia and progressive external ophthalmoplegia (PEO), and are responsible for 5–10% of HSP^34^. HSP is characterized by the degeneration of motor axons of the corticospinal tracts and of sensory axons of the fasciculus gracilis with preservation of the cell bodies. An *SPG7* knockout mouse model recapitulates many characteristics of HSP;^22^ however, the molecular mechanisms underlying diseases associated with mutations in *SPG7* remain largely unknown. To complement some of the deficiencies of vertebrate disease models, we created a *Drosophila* model of HSP by using CRISPR/CAS9 gene targeting to delete the *Drosophila SPG7* homolog. We found that *SPG7*^*del*^ mutants are short-lived and exhibit a variety of behavioral defects. Progressive neuron and muscle degeneration accompany these behavioral defects and dysmorphic mitochondria with electron-dense aggregates were detected in degenerating tissues, indicating that the phenotypes of *SPG7*^*del*^ mutants are caused by mitochondrial dysfunction. Transmission electron microscopy of photoreceptor neurons revealed that the neurodegenerative phenotype of *SPG7*^*del*^ mutants initiates at the synaptic terminal, thus recapitulating one of the hallmark characteristics of HSP. Our fly model provides a foundation for detailed exploration of the molecular mechanisms underlying HSP.

One of the most intriguing phenotypic features of *SPG7*^*del*^ mutant flies is the appearance of abnormally large and dysfunctional mitochondria in the synaptic terminals of photoreceptor cells. These findings also corroborate with the specific pattern of degeneration referred to as “dying-back axonopathy” in HSP, which begins distally at the synaptic terminal and proceeds proximally towards the cell body^35^. Our current findings, coupled with previously published work on *SPG7*^−/−^ mice, lead us to hypothesize that neuropathology in *SPG7*^*del*^ mutants arises from mitochondrial dysfunction that initiates at the synapse and progresses to an axonal trafficking defect, axonal swelling, and ultimately neurodegeneration. Our finding that *SPG7*^*del*^ mutants display increased retrograde mitochondrial transport in segmental nerves offers potential support for this model. Increased retrograde mitochondrial transport was also observed following treatment of cultured neurons with the mitochondrial uncoupling agent Antimycin A1, suggesting that this may represent an early stress response aimed at facilitating the turnover or repair of damaged synaptic mitochondria in the soma^36^. Our model would also potentially explain other genetic forms of HSP that are caused by mutations in genes that would be predicted to impact axonal trafficking, including *SPG10*, which encodes a kinesin heavy chain isoform involved in anterograde transport in axons; *SPG4*, which encodes Spastin, a protein involved in catalyzing microtubule severing; and *SPG20*, which encodes Spartin, a protein involved in endosomal trafficking and microtubule dynamics^37–39^_._

Knockdown of *SPG7* in *Caenorhabditis elegans* results in the accumulation of unfolded proteins and the induction of mitochondrial Hsp60, mtHsp70, and other factors through the UPR^mt^^32^. However, despite the accumulation of electron-dense material in the mitochondrial matrix of *SPG7*^*del*^ mutants, we failed to detect evidence of UPR^mt^ induction. One possible explanation for these discordant findings is that the UPR^mt^ pathway may not be conserved between *Drosophila* and *C. elegans*. For example, the transcription factor Atfs-1 has a central role in the UPR^mt^, but this factor has only been conclusively identified in worms and no homolog of Atfs-1 has been reported in *Drosophila*. It is also possible that a different constellation of chaperones is induced during UPR^mt^ in *Drosophila* to cope with the increased load of misfolded protein aggregates^40,41^. Alternatively, the accumulation of damaged and misfolded proteins in *Drosophila* may activate alternative quality control pathways, including the destruction of mitochondria through PINK1/Parkin-mediated mitophagy, or the degradation of damaged mitochondrial components through a PINK1-Parkin-mediated vesicular pathway^5,42–44^. Further work will be required to distinguish between these models.

Our finding that *SPG7*^*del*^ mutants have reduced complex I abundance and activity agrees with previous work showing that fibroblasts from HSP individuals manifest a complex I assembly defect that results in reduced complex I activity^45^. However, in contrast to findings from HSP fibroblasts, we also detected a significant reduction in the activity of complex II in *SPG7*^*del*^ mutants. Complex II has recently emerged as a regulator of cell death through increased ROS production^46–48^. We propose three alternative hypotheses to explain the decrease in the activity of complex II in *SPG7*^*del*^ mutants. One possibility is that paraplegin is directly involved in the assembly of complex II. Another possibility is that paraplegin promotes the turnover or processing of a factor that influences electron transfer between succinate and ubiquinone-binding subunits. Finally decreased complex II activity in *SPG7*^*del*^ mutants could result from mitochondrial calcium overload. Recent work indicates that Afg3l2 and paraplegin promote the degradation of the essential mitochondrial calcium uniporter regulator (EMRE)^49,50^. Loss of *m*-AAA protease activity results in the constitutive activation of mitochondrial calcium uniporter channel activity and increased Ca^2+^ influx into mitochondria. Mitochondrial Ca^2+^ overload has been implicated in ROS mediated cell death induction by disintegration of complex II^46^. Genetic and pharmacological manipulations designed to offset an increase in cytosolic calcium should enable a direct test of the role of calcium in the phenotypes of *SPG7*^*del*^ mutants.

In summary our fruit fly model of HSP faithfully recapitulates key features of the human disease and provides a genetically tractable system to explore the molecular mechanisms underlying HSP. In previous work, we used a proteomic methodology to identify mitochondrial proteins with altered turnover kinetics in *Drosophila* mutants defective in key mitophagy-promoting and autophagy-promoting genes^51^_._ The application of this methodology to *SPG7*^*del*^ mutants, along with the genetic tools available for gene manipulation in *Drosophila*, should enable rapid identification of paraplegin substrates and evaluation of their roles in pathogenesis. This work should provide important insight into the mechanisms responsible for diseases associated with mutations in *SPG7*, and, ultimately, the development of therapies for these disorders.

## Materials and methods

### Fly stocks and maintenance

All fly stocks and genetic crosses were maintained on standard cornmeal-molasses food at 25 °C, on a 12 h:12 h light-dark cycle. The *w*^1118^, *UAS-CD8-GFP*, *UAS-MITO-GFP*, *Act-gal4*, *D42*-gal4, *CCAP*-gal4, *Da*-gal4, y1 M{vas-Cas9.RFP-}ZH-2A *w*^1118^, and X chromosome duplication stocks Dp(1;3)DC048 and Dp(1;3)DC406 were obtained from the Bloomington Stock Center (Bloomington, IN, USA). The *SPG7*^*del*^ allele was created using CRISPR-CAS9-mediated gene editing in accordance with a published procedure^19^_._ Briefly, our procedure involved replacing the *SPG7* coding sequence with Ds-red through homology-mediated repair. The following primer sequences were used to design guide RNAs targeting the 5′ and 3′ UTR regions of *SPG7*:

5′-Guide RNA

Sense oligo—CTTCGTCGCAGCCGGTCCGCGATT

Antisense oligo—AAACAATCGCGGACCGGCTGCGAC

3′-Guide RNA

Sense oligo—CTTCGCTAATAAGACGCGTCGCGG

Antisense oligo—AAACCCGCGACGCGTCTTATTAGC

To facilitate homology-directed repair, the *pHD-DsRed-attP* vector containing the eye-specific 3xP3 promoter fused with dsRed was appended with sequences flanking the paraplegin coding sequences. The *SPG7* flanking sequences were amplified from genomic DNA using the following primer sequences in PCR:

5′-Homology arm

Forward—CCGGCACCTGCGGCCTCGCATGCGGGTCTCACTCACCTTCACCC

Reverse—CCGGCACCTGCGGCCCTACCGCGGACCGGCTGCGACTACGTAACTCC

3′-Homology arm

Forward—GGCCGCTCTTCATATCGGCGGGCAAGGCGACTTTTAACAACC

Reverse—CCGGGCTCTTCTGACACTGACGGAATCCAGCCAGAAGTCGGG

The 5′ and 3′ homology arms were then cloned into the *pHD-DsRed-attP* vector using *Aar*I and *Sap*I restriction enzymes, respectively. Flies bearing the DsRed marker were identified by screening the injected adults for expression of red fluorescence in the compound eye. The absence of the paraplegin coding sequence was verified by whole-genome sequencing.

For all analyses involving adult flies, age refers to the number of days following eclosion.

### Behavioral and lifespan analyses

All behavioral analyses were performed using male flies. Longevity assays involved 10–15 flies per vial. Food was changed every second day and the number of dead flies was counted. Kaplan–Meier lifespan curves were generated using Microsoft Office Excel, and log-rank test was used to determine statistical significance. For all remaining analyses, flies were anesthetized by briefly exposing them to CO_2_ and allowing them to recover for at least 24 h before the experiment.

Climbing behavior was assessed using the Rapid Iterative Negative Geotaxis (RING) assay at 1 week and 4 weeks of age, according to a previously published protocol^52^. Briefly, flies were transferred into plastic vials and loaded onto the RING apparatus (10–12 flies per vial). The apparatus was gently tapped down 3–4 times to initiate the climbing response and the height climbed by each fly after 4 s was recorded. The climbing assay was repeated three times and the average height climbed in the three trials was calculated.

To conduct the bang sensitivity test, groups of 5–6 flies were transferred into empty plastic vials and vortexed for 10 s at the maximum setting. The time required for flies to recover from paralysis was then recorded. Experiments were repeated with a different cohort of flies each time, and the mean recovery time was calculated.

To analyze heat sensitivity, groups of 5–6 flies were placed in watertight glass vial and vials were then submerged in a water bath preheated to 39 °C for 6 min. The number of paralyzed flies was then recorded following this heat challenge.

Flight assays were performed according to a previously published protocol^20,21^. Briefly, an acetate sheet coated with grease was inserted into a 2-liter graduated cylinder. Flies were tapped into a funnel at the top of the cylinder and the number of flies that fell to the bottom of the cylinder was counted to calculate the fraction of non-fliers after each trial.

### Histological analysis

Histological analyses of brain and muscle tissues were performed as previously described^53^. Briefly, tissues were fixed in Carnoy’s fixation solution (10% acetic acid; 30% chloroform; 60% absolute ethanol) for 3.5 h and dehydrated in ethanol. Infiltration was performed using paraffin at 60 °C, and 4-μm sections were analyzed by hematoxylin and eosin staining. Images were collected on a Nikon Optiphot-2 using a ×10 objective. Brain vacuole size and area was quantified using ImageJ software.

### Transmission electron microscopy

TEM was performed as previously described with some modifications^54^. Briefly, tissues from 28-day-old flies were dissected in fixative containing 2.5% glutaraldehyde, and 2% paraformaldehyde in 0.1 M sodium cacodylate buffer, pH 7.4, and incubated overnight at 4 °C. Fixed tissues were then postfixed in 1% OsO_4_, dehydrated in an ethanol series, and embedded using Epon. Samples were subjected to ultra-thin sectioning at 70 nm and stained with 6% uranyl acetate and a Reynolds lead citrate solution before TEM examination. Grids were viewed using a JEOL JEM 1400 transmission electron microscope.

### Immunofluorescence and confocal microscopy

Thoraces from young (1 week) and old (4 week) flies were dissected in cold PBS buffer, then fixed in 4% paraformaldehyde for 1 h. Dissected tissues were then washed twice in PBS followed by staining with phalloidin-568 (Life Technologies) at a 1:250 dilution for 1 h and imaged using an Olympus FV-1000 confocal microscope with a 60x lens and a 1x digital zoom.

To examine the localization of paraplegin, fixed thoraces dissected from *w*^1118^ as described above were incubated overnight with rabbit anti-paraplegin (1:250) and mouse anti-cytochrome *c* (Cyto C) (1:1000, BD Biosciences) antibody. After washing with PBS (including 0.3% Triton X-100), tissues were incubated overnight with 1:500 anti-rabbit Alexa 488 secondary antiserum and 1:500 anti-mouse Alexa 568 secondary antiserum. Images were acquired sequentially with 488 nm and 561 nm lasers on an Olympus FV-1000 with a 60x lens.

To examine the morphology of neuromuscular junctions, third instar larvae were dissected in PBS buffer followed by fixation in 4% paraformaldehyde. Type 1b synaptic boutons of muscle 4 in abdominal segments 2 and 3- (A2–A3) were visualized using the presynaptic marker anti-HRP-Cy3 (1:200, Jackson Immunoresearch) and the postsynaptic marker anti-discs large 1 (1:50, DSHB).

To examine axonal morphology, legs from flies bearing the *UAS-CD8-GFP* and *D42- GAL4* transgenes were dissected, and GFP fluorescence was visualized using confocal microscopy at 10x magnification. At least 20 legs from 10 different flies were imaged.

### Mitochondrial respiratory chain activity assay

Mitochondrial fractions for respiratory chain activity assays were prepared using a published procedure with minor modifications^55^. Briefly, 800–900 flies were homogenized in 5 mM HEPES (pH 7.5), 210 mM mannitol, 70 mM sucrose, and 1 mM EGTA. The lysate was subjected to centrifugation at 3500 rpm for 5 min to remove cuticle and cellular debris. The supernatant was then subjected to centrifugation a second time at 15,000 rpm for 20 min to isolate the mitochondrial pellet. Mitochondria were resuspended in 250 mM sucrose, 2 mM EDTA, 100 mM Tris–HCl, pH 7.4, flash frozen in liquid nitrogen, and then stored at −80 °C.

Respiratory chain activity assays were performed as previously described with minor modifications^55^. Complex I activity was measured by monitoring the oxidation of NADH at 340 nm using ubiquinone-1 as an electron acceptor in a buffer containing 50 mM potassium phosphate (pH 7.5), 0.1 mM NADH, and 0.3 mM potassium cyanide. Background activity was determined using 10 μM rotenone, and used to calculate complex I-specific activity. Complex II activity was measured by monitoring the reduction of 2,6- dichlorophenolindophenol at 600 nm in a reaction mixture of 25 mM potassium phosphate (pH 7.5), 20 mM succinate, 80 μM 2,6-dichlorophenol-indophenol, 50 μM Decylubiquinone, and 0.3 mm potassium cyanide. Background activity was determined using 10 mM malonate, and used to calculate complex II-specific activity. Complex III activity was determined by monitoring the reduction of cytochrome *c* at 550 nm in a reaction mixture of 25 mM potassium phosphate (pH 7.5), 100 μM reduced decylubiquinone, 75 μM cytochrome *c* and 0.5 mM potassium cyanide. Background activity was calculated using antimycin A (10 μg ml^−1^), and used to calculate complex III-specific activity. Complex IV activity was performed by monitoring the oxidation of cytochrome *c* at 550 nm in a reaction mixture of 25 mM potassium phosphate (pH 7.5), and 0.05 mM reduced cytochrome *c*. Background activity was determined using 0.3 mM potassium cyanide, and used to calculate complex IV-specific activity. All activities were normalized against citrate synthase activity, which was determined by following the reduction of 5,5′-dithiobis(2-nitrobenzoic acid) at 412 nm in presence of acetyl-coenzyme A and oxaloacetate. Approximately 10–40 μg of mitochondria was used in all assays.

### Blue native PAGE (BN-PAGE) analysis

BN-PAGE was performed according to a previously published protocol^56^. Briefly, 50 µg of mitochondria prepared from 1 week and 4 week old adult flies as solubilized in a buffer containing a digitonin:protein (mass:mass) ratio of 8 and subjected to centrifugation at 20,000×*g* for 10 min at 4 °C. The supernatent was treated with coomassie G-250 and used in Native PAGE analysis. Complex I assembly was analyzed using an anti-NDUFS3 antiserum.

### Subcellular fractionation

Subcellular fractionation was performed as previously described^57^. Briefly, a crude mitochondrial pellet was obtained as described above and subsequently resuspended in buffer containing 0.27 M d-mannitol, 0.01 M Tris-base, and 0.1 mM EDTA and carefully overlaid on a sucrose gradient prepared by combining 1 ml of 1.7 M sucrose with 1.6 ml of 1 M sucrose. Samples were then subjected to ultracentrifugation at 40,000×*g* for 22 min and gradient fractions were recovered using a 1-ml syringe with a 20-G needle.

### Paraquat sensitivity test

Two hundred flies were starved for 6 h in plastic vials containing filter paper soaked with water (10–15 flies per vial). Flies were then transferred to experimental vials containing filter paper soaked with 10 mM paraquat in 5% sucrose solution, or control vials containing filter paper soaked with 5% sucrose solution alone. Flies were transferred to new vials daily. The number of dead flies was counted daily over a period of 1 week.

### Immunoblotting

Whole flies were homogenized in RIPA buffer and the supernatant was subjected to SDS-PAGE electrophoresis. Following electrophoresis, proteins were transferred to PVDF membrane, and the membrane was blocked with 5% milk, then incubated overnight with antisera. Antibodies were used at the following concentrations for immunoblots: COX IV (ab33985, Abcam), 1:5000; NDUFS3 (ab14711, Abcam), 1:1000; SDHB (ab14714, Abcam), 1:1000; phospho-EIF2α (ab32157, Abcam), 1:1000; EIF2α (ab26197, Abcam), 1:1000; Lon (NBP1-81734, Novus Biologicals), 1:5000; Hsp60 (4870S, Cell Signaling Technology), 1:1000; mtHsp70 (sc-13967, Santa Cruz Biotechnology), 1:5000; ATP Synthase β (A21351, Thermo Fisher Scientific), 1:1000; and Actin (MAB1501, Chemicon), 1:10,000. Chemiluminescence was used for antibody detection and western blot images were quantified using ImageJ software and normalized to Actin. Each experiment was performed at least three times.

### Analysis of mitochondrial transport

Mitochondrial trafficking was measured in third instar larvae expressing *UAS-MITOGFP* driven by the *CCAP-Gal4* driver^58,59^. Briefly, larvae were pinned with the dorsal surface facing upward and dissected in a buffer containing 128 mM NaCl, 5 mM EGTA, 4 mM MgCl_2_, 2 mM KCl, 5 mM HEPES and 36 mM sucrose, pH adjusted to 7.2. A small incision was made at the posterior end and continued along the dorsal midline. After removing internal organs, larvae were transferred to a chamber on a glass side constructed with the aid of double sided tape and imaged using an Olympus FV-1000 fluoview confocal microscope with a 60x water immersion lens. Images were captured at a rate of 1 frame per 3.25 s for 100 frames. One axon was analyzed per larvae and a total of 18 axons for control and 19 axons for *SPG7*^*del*^ mutants were analyzed. Kymographs were constructed using the KymographBuilder plugin in ImageJ software, and analyzed as described previously^58,59^.

### Statistics

All data is represented as mean ± s.e.m. Unless otherwise stated, statistical significance tests were calculated using an unpaired two-tailed Student’s *t*-test in GraphPad Prism 7.

## Electronic supplementary material

SUPPLEMENTAL MATERIAL

Supplementary Movie 1

Supplementary Movie 2
