# Supplementary material for: Loss of the Drosophilam-AAA mitochondrial protease paraplegin results in mitochondrial dysfunction, shortened lifespan, and neuronal and muscular degeneration
Source: Cell Death Dis. 2018 Feb 21;9(3):304. doi: 10.1038/s41419-018-0365-8 (PMC5833341; doi:10.1038/s41419-018-0365-8)
Supplement: Supplementary file 1 — SUPPLEMENTAL MATERIAL [file 41419_2018_365_MOESM1_ESM.docx]

***D. Melanogaster*** ---MLKGLRHVRNALQRGGSQLIQL--RSATGQPPRIGRELSLL--------------QL

***H. Sapiens*** MAVLLLLLRALRRGPGPGPRPLWGPGPAWSPGFPARPGRGRPYMASRPPGDLAEAGGRAL

:* ** :*.. * * : * * * ** : *

***D. Melanogaster*** SRRQVMQLQAEYKAIVGLIARSLQLTPKEVRLMHLRSLSSTAKPPTASDGKTEKHEQPEK

***H. Sapiens*** QSLQLRLLTPTFEGINGLLLKQH-LVQNPVRLWQLLGGTFYFN--T------SRLKQKN-

. *: * ::.* **: :. *. : *** :* . : : * .: :* :

***D. Melanogaster*** GSAKAKDDSSKEKKSAVAAEDGDKSSRAAGEEAASTPTSSSTIGEPGEDP-NKNSNENDE

***H. Sapiens*** ---KEK----------------DKSK---G-------------KAPEEDEEERRRRERDD

* * ***. * * ** ::. .*.*:

***D. Melanogaster*** KMRSVLTKAVLWLFTIYMFVAFFSLLITPRSERPEGSTRYVSWNEFVHHMLAVGEVKELI

***H. Sapiens*** QMYRERLR---TLLVIAVV---MSLL-----NALSTSGGSISWNDFVHEMLAKGEVQRVQ

:* : *:.* :. :*** : . * :***:***.*** ***:.:

***D. Melanogaster*** IRPDMEMVTIILHEGAVIKGRKVSSTIFHMAVADANKFEEKLRDVEKRLGI--KDGVPVT

***H. Sapiens*** VVPESDVVEVYLHPGAVVFGRPRLALMYRMQVANIDKFEEKLRAAEDELNIEAKDRIPVS

: *: ::* : ** ***: ** : :::* **: :******* .*..*.* ** :**:

***D. Melanogaster*** YDRQTDTTGRILMLLLV---CALLMSIATRMKSIKSPLSMDSFNQMGRAKFTLVDPFDGG

***H. Sapiens*** YKRTGFFGNALYSVGMTAVGLAILWYVFRLAGMTGREGGFSAFNQLKMARFTIVDGK-MG

*.* . : : :. *:* : .:.:***: *:**:** *

| ***D. H.*** | ***Melanogaster***  ***Sapiens*** | RGVLFRDVAGLSEAKQEVKEFVDYLKSPEKYQRLGAKVPRGALLLGPPGCGKTLLAKAVA KGVSFKDVAGMHEAKLEVREFVDYLKSPERFLQLGAKVPKGALLLGPPGCGKTLLAKAVA |
| --- | --- | --- |
|  |  | :** *:****: *** **:**********:: :******:******************** |
| ***D. H.*** | ***Melanogaster***  ***Sapiens*** | TEAQVPFLSMNGSEFIEMIGGLGAARVRDLFKEGKKRAPCIIYIDEIDAIGRQRSGTESM TEAQVPFLAMAGPEFVEVIGGLGAARVRSLFKEARARAPCIVYIDEIDAVGKKRSTTM-- |
|  |  | ********:* * **:*:**********.****.: *****:*******:*::** * |
| ***D. H.*** | ***Melanogaster***  ***Sapiens*** | GQGSSGESEQTLNQLLVEMDGMATKEGVLMLASTNRADILDKALLRPGRFDRHILIDLPT SGFSNTEEEQTLNQLLVEMDGMGTTDHVIVLASTNRADILDGALMRPGRLDRHVFIDLPT |
|  |  | . *. *.**************.*.: *::*********** **:****:***::***** |
| ***D. H.*** | ***Melanogaster***  ***Sapiens*** | LAERKEIFEKHLSSVKLESPPTTFSQRLARLTPGFSGADIANVCNEAALHAARNTQMEVS LQERREIFEQHLKSLKLTQSSTFYSQRLAELTPGFSGADIANICNEAALHAAREGHTSVH |
| ***D.*** | ***Melanogaster*** | * **:****:**.*:** . * :*****.************:**********: : .* SKNLEYAVERLVGGTEKRSHALSLAERKVIAYHESGHALVGWMLPNSDILLKVTIVPRTS |
| ***H.*** | ***Sapiens*** | TLNFEYAVERVLAGTAKKSKILSKEEQKVVAFHESGHALVGWMLEHTEAVMKVSITPRTN |
|  |  | : *:******::.** *:*: ** *:**:*:************ ::: ::**:*.***. |
| ***D. H.*** | ***Melanogaster***  ***Sapiens*** | LALGFAQYTPSEQHLYSKEELFDKMCMALGGRAAENLVFNRITTGAQNDLEKVTKIAYSQ AALGFAQMLPRDQHLFTKEQLFERMCMALGGRASEALSFNEVTSGAQDDLRKVTRIAYSM |
|  |  | ****** * :***::**:**::*********:* * **.:*:***:**.***:**** |
| ***D. H.*** | ***Melanogaster***  ***Sapiens*** | IKKFGMNDTLGPIYVRDADETEGGGAMGSGGKKPFSRAMESMIDNEARHVVASAYQTTEG VKQFGMAPGIGPISFPEAQEG----LM-GIGRRPFSQGLQQMMDHEARLLVAKAYRHTEK |

:*:*** :*** . :*:* * . *::***:.::.*:*:*** :**.**: **

***D. Melanogaster*** ILTTHRDKLEKLAEALLEKETLDYDQVVQLIGPPPYDLGKRQVESVEFEQS--LKNLSTD

***H. Sapiens*** VLQDNLDKLQALANALLEKEVINYEDIEALIGPPPHGPKKMIAPQRWIDAQREKQDLGEE

:* : ***: **:******.::*::: ******:. * . . :: . ::*. :

***D. Melanogaster*** -TDATKA------------

***H. Sapiens*** ETEETQQPPLGGEEPTWPK

*: *:

**Supplementary Figure S1A:** Sequence alignment of *Homo sapiens* paraplegin and *Drosophila melanogaster* paraplegin using the clustal omega algorithm. **‘*’** denotes the identical ‘:’ denotes strongly similar and ‘.’ denotes weakly similar amino acid residue.

**AAA**

**FtsH-**

**extracellular**

**169**

**278**

**377**

**513**

**575**

**785**

**Peptidase M41**

1

819

**Supplementary Figure S1B:** Domain organization of paraplegin using Pfam motif prediction algorithm highlighting the presence of FtsH-extracellular, AAA and metallopeptidase M41 domain.


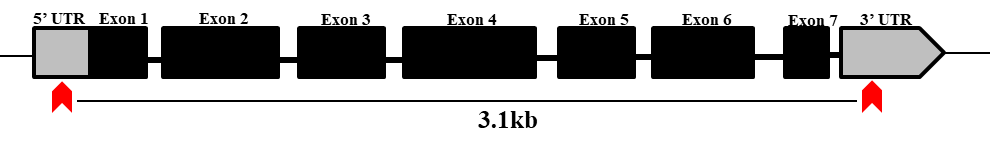


**Exon 1**

**Exon 2**

**Exon 3**

**Exon 4**

**Exon 5**

**Exon 6**

**Exon 7**

**3’ UTR**

**5’ UTR**

**3.1kb**

**Supplementary Figure S2:** The CG2658 gene consists of 7 exons (black boxes). Red arrowheads highlight the region targeted by guide RNAs for CRISPR mediated deletion of *SPG7*.

**Supplementary Figure S3:** (a) Schematic representation of autosomes containing duplications spanning the *SPG7* gene CG2658. (b) Shortened lifespan of *SPG7^del^* flies was rescued by both duplications spanning the *SPG7* gene. The genotypes of flies used are as follows and the number of flies used is indicated within parentheses - *w*^1118^ (N = 207), *SPG7^del^* (N = 179), *SPG7^del^*; Dp(1;3)DC048 (N = 187) and *SPG7^del^*; Dp(1;3)DC406 (N = 195). Significance was determined using a log-rank test (****p < 0.0005). (c) Locomotor performance of 4-weeks old *SPG7^del^* flies was rescued by duplications spanning the *SPG7* gene. (N = 60 for each genotype, ****p < 0.0005 by one-way ANOVA followed by tukey’s test).


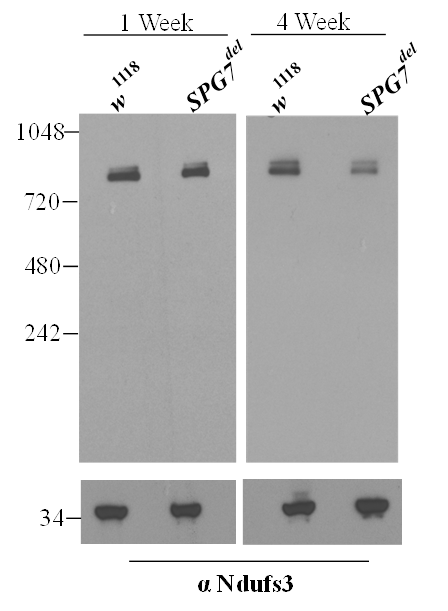


**Supplementary Figure S4:** Results of Blue Native gel analysis and immunoblotting to analyze complex I abundance. Immunoblotting was performed using antisera to the Ndufs3 protein. Because our data in Figure 6 shows that the abundance of Ndufs3 is not influenced in *SPG7^del^* mutants, we performed western blot analysis using the anti-Ndufs3 antisera to normalize protein loading in the Blue Native gel.


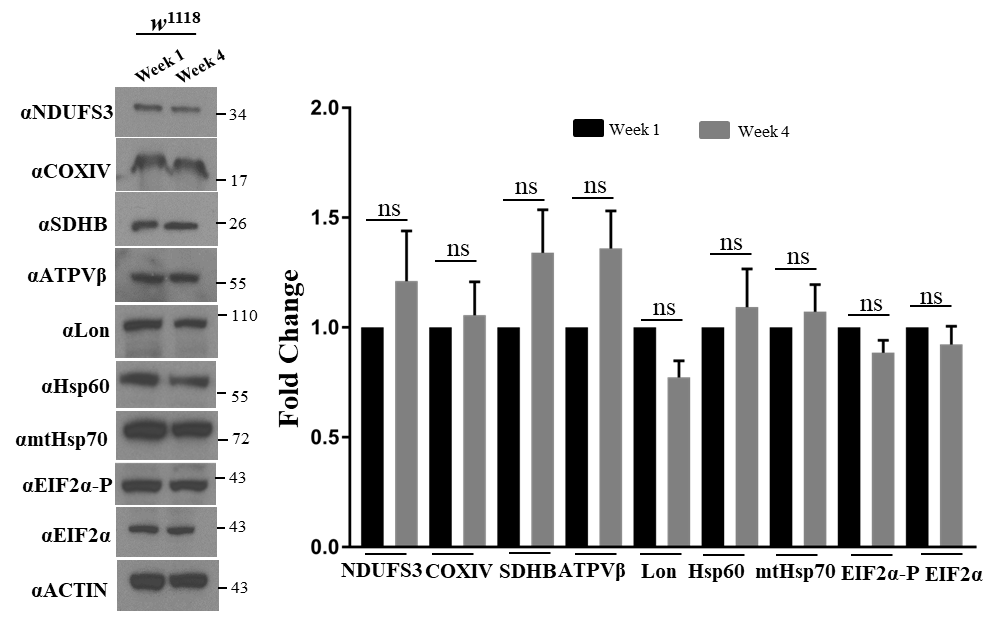


**Supplementary Figure S5:** Immunoblot analysis of the indicated respiratory chain complex subunits and UPR^mt^ markers in young (1-week) and old (4-weeks) *w*^1118^ controls. Right panel highlights the fold change in abundance after normalizing using actin as a loading control. Significance was determined using Student's t-test.
